# SLCO1B1 and SLC19A1 Gene Variants and Irinotecan-Induced Rapid Response and Survival: A Prospective Multicenter Pharmacogenetics Study of Metastatic Colorectal Cancer

**DOI:** 10.1371/journal.pone.0077223

**Published:** 2013-10-15

**Authors:** Liu Huang, Tao Zhang, Conghua Xie, Xin Liao, Qianqian Yu, Jueping Feng, Hong Ma, Jing Dai, Min Li, Jigui Chen, Aihua Zang, Qian Wang, Shuwang Ge, Kai Qin, Juan Cai, Xianglin Yuan

**Affiliations:** 1 Department of Oncology, Tongji Hospital, Tongji Medical College, Huazhong University of Science and Technology, Wuhan, P. R. China; 2 Cancer Center of Union Hospital, Tongji Medical College, Huazhong University of Science and Technology, Wuhan, P. R. China; 3 Department of Radiation and Medical Oncology, Zhongnan Hospital, Wuhan University, Wuhan, P. R. China; 4 Department of Oncology, Wuhan Pu-Ai Hospital, Tongji Medical College, Huazhong University of Science and Technology, Wuhan, P. R. China; 5 Department of Surgery, Wuhan 8^th^ Hospital, Wuhan, P.R. China; 6 Hubei Cancer Hospital, Wuhan, P. R. China; 7 Department of Nephrology, Tongji Hospital, Tongji Medical College, Huazhong University of Science and Technology, Wuhan, P. R. China; 8 Department of Oncology, Yijishan Hospital, Wannan Medical College, Wuhu, P. R. China; Sudbury Regional Hospital, Canada

## Abstract

**Background:**

Rapid response to chemotherapy in metastatic colorectal cancer (mCRC) patients (response within 12 weeks of chemotherapy) may increase the chance of complete resection and improved survival. Few molecular markers predict irinotecan-induced rapid response and survival. Single-nucleotide polymorphisms (SNPs) in solute carrier genes are reported to correlate with the variable pharmacokinetics of irinotecan and folate in cancer patients. This study aims to evaluate the predictive role of 3 SNPs in mCRC patients treated with irinotecan and fluoropyrimidine-containing regimens.

**Materials and Methods:**

Three SNPs were selected and genotyped in 137 mCRC patients from a Chinese prospective multicenter trial (NCT01282658). The chi-squared test, univariate and multivariable logistic regression model, and receiver operating characteristic analysis were used to evaluate correlations between the genotypes and rapid response. Kaplan-Meier survival analysis and Cox proportional hazard models were used to evaluate the associations between genotypes and survival outcomes. Benjamini and Hochberg False Discovery Rate correction was used in multiple testing

**Results:**

Genotype GA/AA of SNP rs2306283 of the gene *SLCO1B1* and genotype GG of SNP rs1051266 of the gene *SLC19A1* were associated with a higher rapid response rate (odds ratio [OR] =3.583 and 3.521, 95%CI =1.301-9.871 and 1.271-9.804, *p*=0.011 and p=0.013, respectively). The response rate was 70% in patients with both genotypes, compared with only 19.7% in the remaining patients (OR = 9.489, 95%CI = 2.191-41.093, Fisher's exact test *p*=0.002). Their significances were all maintained even after multiple testing (all *p*_*c*_ < 0.05). The rs2306283 GA/AA genotype was also an independent prognostic factor of longer progression-free survival (PFS) (hazard ratio = 0.402, 95%CI = 0.171-0.945, *p*=0.037). None of the SNPs predicted overall survival.

**Conclusions:**

Polymorphisms of solute carriers’ may be useful to predict rapid response to irinotecan plus fluoropyrimidine and PFS in mCRC patients.

**Trial Registry:**

ClinicalTrials.gov NCT01282658

http://www.clinicaltrials.gov/ct2/show/NCT01282658

## Introduction

Colorectal cancer (CRC) is one of the leading causes of cancer death in the United States and China[1,2]. Complete resection of all known involved sites in selected metastatic colorectal cancer (mCRC) patients significantly improves their survival[3]. irinotecan plus fluoropyrimidine (± leucovorin) is one of the key regimens for mCRC treatment and can help to convert an unresectable patient to a resectable status[4]. However, only 30-50% of patients respond to first-line irinotecan plus fluoropyrimidine chemotherapy[5,6]. To date, conversion and neoadjuvant treatment is usually limited to 2 to 3 months. Response within 12 weeks to chemotherapy greatly improves the chance of complete resection and longer survival for mCRC patients. Therefore it is of great significance to identify patients who will respond to the relevant chemotherapy within 12 weeks. 

Some germline polymorphisms of metabolizing enzymes of 5-fluorouracil (5-FU) and irinotecan have been described to correlate with the degree of toxicity in CRC patients. However, clinical data do not unequivocally support their influence on cancer response till now. Only limited data is available to predict rapid response to guide treatment choice [4,7-13].. 

Solute carriers account for variable pharmacokinetics of irinotecan and folate in cancer patients. For example, solute carrier organic anion transporter family member 1B1 (*SLCO1B1*) is a major influx transporter expressed on the basolateral membrane of human hepatocytes that mediates 7-ethyl-10-hydroxy-camptothecin (SN38, the active metabolite of irinotecan) disposition[14]. A *SLCO1B1* single-nucleotide polymorphism (SNP), rs4149056 (521T>C), has been demonstrated to be associated with a higher area under the concentration-time curve of SN38 (AUC_SN38_) and grade ≥ 3 neutropenia in lung cancer patients treated with irinotecan and cisplatin[15-17]. However, evidence for an association between *SLCO1B1* SNPs and irinotecan-related tumor response and survival in mCRC patients is still unclear.

The human solute carrier family 19, member1 (*SLC19A1*) gene encodes reduced folate carrier protein 1 (RFC1), which mediates intracellular uptake of folate-[18,19]. Previous studies identified that human colon cancer cell lines kept in high-folate medium showed a lower sensitivity to fluorouracil (5-FU) alone or 5-FU plus leucovorin (an active form of folate) than the same cell lines kept in low-folate medium [20]. However, there have been few studies focusing on the relationship of *SLC19A1* gene variants with inter-patient variation in combined irinotecan and fluoropyrimidine regimens (FOLFIRI [irinotecan plus 5-FU and leucovorin] / mCapeIRI [irinotecan plus capecitabine]).

There is no doubt that non-genetic covariate controls are very important for understanding the contribution of genetic variation in pharmacogenetic studies. Here, we conducted a prospective multi-center study in mCRC patients to investigate whether SNPs in solute carrier genes was associated with rapid tumor response to FOLFIRI/mCapeIRI and improved survival.

**Table 1:** Patients’ characteristics.

	***n***	**%**
**No. of patients**	137	100
**Age, years**		
**Median (range**)	53 (18–75)	
**Sex**		
Male	87	63.5
Female	50	36.5
**KPS**		
60%	16	11.7
70%	44	32.1
≥80%	77	56.2
**Smoker**		
No	84	61.3
Yes	53	38.7
**Chemotherapy regimen**		
FOLFIRI	104	75.9
mCapeIRI	33	24.1
**Primary tumor**		
Colon	73	53.3
Rectum	64	46.7

KPS, Karnofsky's index of performance status; FOLFIRI, irinotecan plus 5-FU and leucovorin; mCapeIRI, irinotecan plus capecitabine.

## Materials and Methods

### Patient eligibility and study design

This study was approved by the Ethics Committee of Huazhong University of Science and Technology on 12 November 2010 and registered on http://www.clinicaltrials.gov with the reference number NCT01282658. Six cancer centers in Hubei province were involved (Table S1 in File S2). The study was coordinated and sponsored by the Department of Oncology, Tongji Hospital, Tongji Medical College, Huazhong University of Science and Technology, Wuhan, China. All participating institutions approved the study protocol. Written informed consent was obtained from each patient before recruitment. Peripheral blood samples were obtained from patients who agreed to provide blood. 

**Figure 1:**
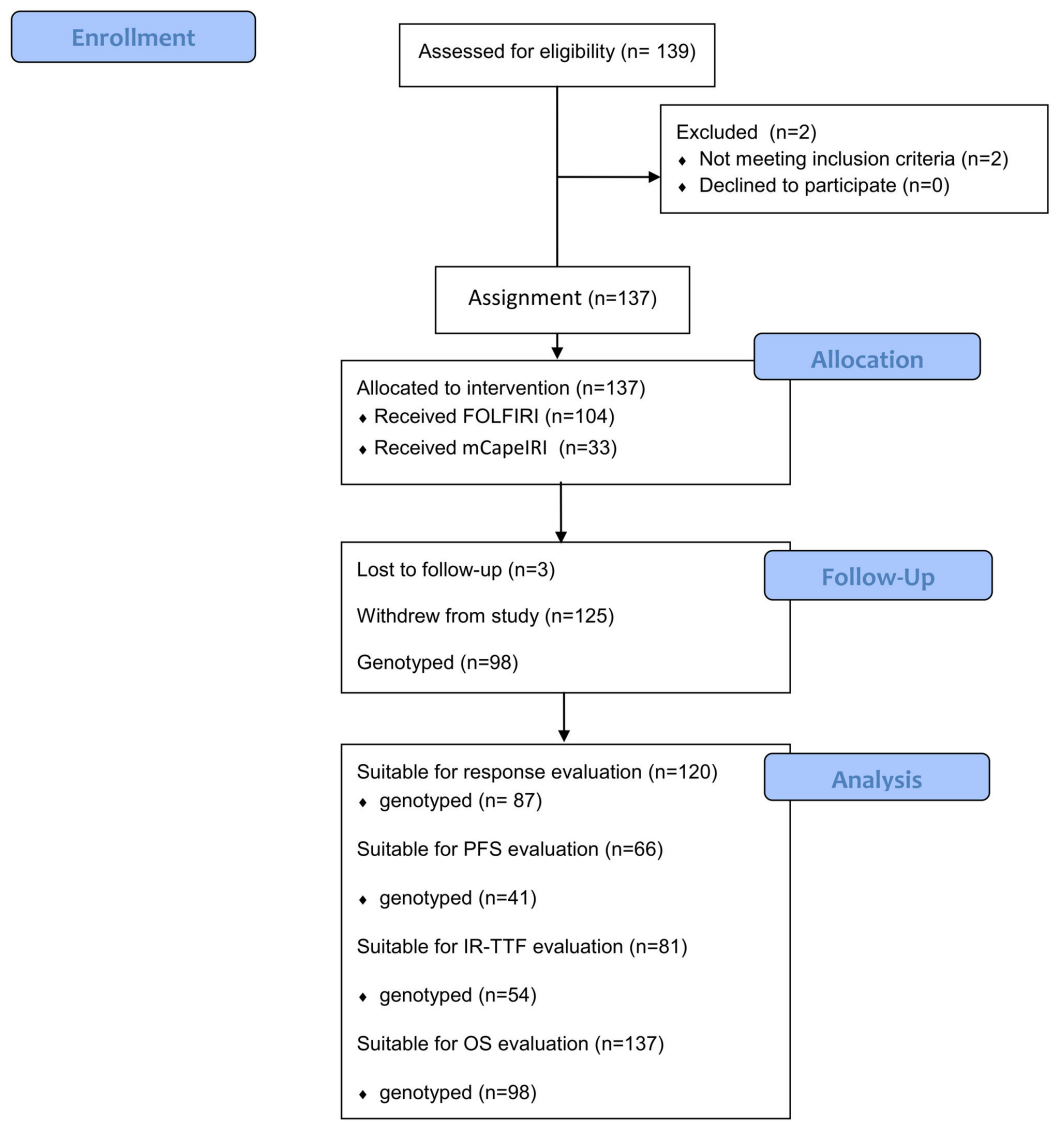
Flow chart.

We choose type I error α = 0.05, 1-β = 0.8, two-sided test, provided the target SNP allele frequency in the population was about 20%, treatment efficacy was about 30%, OR ≥ 3.5, the calculated samples size was 86 by Quanto (Version 1.2.4).

Eligibility criteria included histologically confirmed unresectable metastatic adenocarcinoma of the colorectum; age between 18 and 75 years old; measurable disease, defined according to the Response Evaluation Criteria In Solid Tumors version 1.1 (RECIST1.1)[21]; no previous irinotecan exposure; no expected course of radiotherapy during first-line chemotherapy; Karnofsky's index of performance status ≥ 60 or Eastern Cooperative Oncology Group Performance Status Scale ≤ 2; patients not pregnant or nursing; patients voluntarily signed the informed consent; total bilirubin ≤ 1.5 times the upper limit of normal (ULN); aspartate aminotransferase and alanine aminotransferase ≤ 2.5 times ULN (≤ 5 times ULN if liver metastases present); creatinine clearance > 50 ml/min or serum creatinine ≤ 1.5 times ULN.

The primary objectives were to assess the correlation between genetic variants and the rapid response rate (RRR) in Chinese mCRC patients. Rapid response was defined as at least a 30% decrease in the sum of the longest diameter of target lesions to the first 12 weeks of chemotherapy. Secondary objectives included the relationship between gene variants and progression-free survival (PFS), irinotecan-related time to treatment failure (IR-TTF), and overall survival (OS). PFS was deﬁned as the time elapsed between the first day of irinotecan treatment and disease progression (PD) or death from any cause, whichever occurred ﬁrst. IR-TTF was calculated from the start of irinotecan treatment to its discontinuation for reasons including PD, intolerable toxicity, or death. OS was calculated as the time from irinotecan treatment initiation until death from any cause or the date of last follow-up[22].

**Table 2:** SNPs information and genotypic frequencies in 203 gastrointestinal cancer and 98 mCRC patients.

**SNPs**	**Gene**	**Allelic change**	**Function**	**AA change**	**Call rate *n*(%)**	**HWE*p***	**MAF**	**Genotype frequency, *n*(%**)		
								**wt/wt**	**wt/var**	**var/var**
**rs1051266**	*SLC19A1*	A>G	M	H27R	201(99.0)	0.30	0.48	57(28.4)	93(46.2)	51(25.4)
					97(99.0)	0.66	0.44	18(18.6)	50(51.5)	29(29.9)
**rs2306283**	*SLCO1B1*	A>G	M	N130D	200(98.5)	0.93	0.24	12(6.0)	73(36.5)	115(57.5)
					97(99.0)	0.36	0.22	3(3.1)	36(37.1)	58(59.8)
**rs4149056**	*SLCO1B1*	T>C	M	V174A	203(100.0)	0.80	0.13	153(75.4)	46(22.7)	4(1.9)
					98(100.0)	0.81	0.13	74(75.5)	22(22.4)	2(2.1)

SNPs, Single-nucleotide polymorphisms; AA, amino acid; mCRC, metastatic colorectal cancer; HWE, Hardy-Weinberg equilibrium; MAF, minor allele frequency; wt, wild type; var, variant; M, missense.

The protocol for this trial and supporting TREND checklist are available as supporting information; see Protocol S1 and Checklist S1.

### Efficacy and toxicity assessment

Patients stayed in hospital for the 3-day chemotherapy. Efficacy was evaluated with a consistent imaging technique (magnetic resonance imaging or computed tomography scan) every 6 weeks by the RECIST 1.1. Toxicity information was collected by face-to-face questionnaires and was graded using the National Cancer Institute Common Toxicity Criteria of Adverse Events version 4.0 at every cycle. A single cycle of chemotherapy was considered enough to evaluate toxicity; otherwise, at least three cycles (6 weeks) of chemotherapy would be needed in the response assessment analysis. Earlier response evaluation was only allowed when patients had severe symptoms indicating progression. Blood counts and biochemistry tests were performed within 72 hours of the beginning of each cycle and 7–10 days after each cycle. Evaluations were performed blinded to the genetic results and were assessed independently by two doctors. A third doctor resolved inconsistencies. The clinical data were monitored by the study sponsor.

### Treatment

The patients who accepted FOLFIRI received, as recommended by the guidelines of the National Comprehensive Cancer Network, irinotecan (Camptosar; Pfizer, Sydney, Australia) 180 mg/m^2^ intravenous (IV) infusion over 30–90 minutes, day 1; leucovorin 400 mg/m^2^ IV infusion to match the duration of irinotecan infusion, day 1; 5-FU 400 mg/m^2^ IV bolus, day 1; then 1200 mg/m^2^/day × 2 days (total 2400 mg/m^2^ over 46–48 hours) continuous infusion; this was repeated every 14 days. Patients who declined continuous infusion were later changed to an mCapeIRI[23] regimen (irinotecan 125 mg/m^2^, days 1 and 8; capecitabine 825–1000 mg/m^2^, twice daily on days 1–14; repeat every 21 days). We modified the standard irinotecan plus capecitabine (CapeIRI[24] or XELIRI[25]) regimen because of our experience in toxicity control. All the patients accepted 5-hydroxytryptamine receptor antagonist (5 mg once a day) 30–60 minutes before irinotecan. The criteria for irinotecan dose reduction are given in Table S2 in File S2. The chemotherapy continued until disease progressed or intolerable toxicities came out or patients asked to withdrew due to any reason. 

### Genotyping

We searched the National Center for Biotechnology Information (NCBI) SNP database (dbSNP; http://www.ncbi.nlm.nih.gov/snp/) and related literature to identify functional SNPs from the *SLCO1B1* and *SLC19A1* genes. The criteria for SNP selection were as follows:. (1) With a minor allele frequency of more than 0.15 in Asian population, (2) Genotype call rate ≥ 95%, (3) Missense SNP. Three SNPs (*SLCO1B1* rs2306283 and rs4149056; and *SLC19A1* rs1051266) were selected for genotyping. Genomic DNA was extracted from peripheral blood using a FUJI whole blood DNA kit (Fujifilm Corporation, Tokyo, Japan). Primers were designed by Genotyping Tools and MassARRAY Assay Design software (version 3.0, Sequenom Inc., San Diego, California). SNPs were genotyped using the Sequenom MassARRAY iPLEX platform. Data were processed and analyzed by Sequenom MassArray TYPER 4.0 software. Details of PCR reactions and primer sequences are available in File S1 and Table S3 in File S2 respectively. Five percent of the samples were randomly selected and genotyped by direct sequencing, with a resulting concordance rate of 100%. Call rate threshold was set at least 95% for each SNP. Hardy-Weinberg equilibrium (HWE) was tested through χ^2^ test and *p* < 0.05 indicated deviation from the equilibrium.

### Statistical analysis

Every variant was evaluated for association with every endpoint. Correlations between RRR and genotypes were tested using the Pearson chi-squared test. The multivariable logistic regression model (Enter) was used to adjust for potential covariates (sex and smoking status as dichotomous variables; age, surface area, and performance status as continuous variables; rs1051266, rs2306283 and rs4149056 genotypes as dichotomous variables). Smoking significantly lowered the exposure to irinotecan, indicating a potential risk of treatment failure in a case-control study[26]; therefore, we accounted for smoking status in the multivariate analysis. Odds ratios (OR) and their 95% confidence interval (CI) were calculated as estimates of the correlations. Receiver operating characteristic (ROC) curves were generated to compare the models with and without positive response predictors. Kaplan-Meier analysis and log-rank test were performed to estimate the distribution of PFS, IR-TTF, and OS and to compare differences between survival curves. To evaluate the relationship between genotypes and PFS, IR-TTF, and OS, multivariate Cox regression (Enter) was performed, adjusting for potential confounding covariates. Hazard ratios (HR) and their 95% CI were calculated as estimates of the correlations. A value of *p* < 0.05 was considered statistically significant in a two-tailed test. Corrected P-value (*p*_*c*_) was obtained by Benjamini and Hochberg False Discovery Rate correction in multiple testing. Haploview software (version 4.2) was used to calculate Linkage disequilibrium (LD) between polymorphisms and perform the analysis of inferred haplotypes. Statistical analysis was performed using SPSS 16.0 statistical software (SPSS Inc., Chicago, IL). 

## Results

### Patient characteristics and treatment outcome

The patients’ characteristics are presented in Table 1. From November 2010 to June 2012, 139 patients were enrolled. Two patients were found to be ineligible according to the monitoring committee evaluation, and were excluded from the final analysis. 

Flow chart was shown in Figure 1. Response evaluation was available for 120 patients. Seventeen patients could not be evaluated for response because of: early cessation of chemotherapy (fewer than three cycles) due to insufferable toxicity (*n* = 5) or to non-medical reasons (*n* = 6); still under observation for response (*n* = 4); or other anti-cancer therapy interfering with the efficacy assessment (*n* = 2). 

The RRR and toxicities are shown in Table S4 and S5 in File S2. Among the 120 patients who were considered suitable for response evaluation, 33 patients (27.5%) achieved a rapid response, of them, 13 patients (39.4%) responded within the first 6 weeks of chemotherapy, and 20 patients (60.6%) responded within 12 weeks of the start of chemotherapy (Table S4 in File S2). Ninety eight patients provided blood samples and were genotyped, and the response was assessable in 88.8% (*n* = 87). No difference for RRR was found between genotyped and non-genotyped patients (26.4% vs. 30.3%, p = 0.672; Pearson chi-squared test).

Thirty-three patients (24.1%) accepted the mCapeIRI regimen. RRR (27.6% vs. 27.5%, *p* = 0.990; Pearson chi-squared test) and survival (Figure S1 in File S3) did not show any differences between the mCapeIRI and FOLFIRI groups. So we did not carry out subgroup analysis according to different chemotherapy regimens. 

**Table 3:** RRR in patients according to genotypes (Pearson chi-squared test).

	**N**	**Patients with RRR n(%)**	**OR (95%CI)**	***p***	***P_c_***
**rs2306283^a^**					
GG	51	8(15.7)			
GA/AA	35	14(40.0)	3.583(1.301-9.871)	0.011	0.022
**rs1051266^a^**					
GG	24	11(45.8)	3.521(1.271-9.804)		
GA/AA	62	12(19.4)		0.013	0.017
**rs4149056^b^**					
TT	67	18(26.9)			
CT/CC	20	5(25.0)	0.907(0.288-2.858)	0.868	0.868
**rs2306283 (GA/AA**)** + rs1051266 (GG**)	10	7(70.0)	9.489(2.191-41.093)		
**others**	76	15(19.7)		0.002^※^	0.008

RRR, rapid response rate; OR, odds ratio; CI, confidence interval; ^※^ Fisher's exact test;

N, No. of assessable patients; ***p*_*c*_**, P-value corrected by Benjamini and Hochberg False Discovery Rate correction.

a genetic model is REC.

b genetic model is DOM.

### SNPs information, genotypic and haplotypic frequencies and LD analysis

In addition to our multi-center cohort, we genotyped SNPs in 203 Chinese gastrointestinal cancer patients (98 mCRC patients and 105 gastric cancer patients). The genotype frequencies are reported in Table 2. The minor allele frequency (MAF) of each SNP was comparable to previously reported data in the NCBI database, except for rs2306283, for which the MAF was lower in our population[27,28] than previously reported. All variants were in HWE (*p* > 0.05).

**Figure 2:**
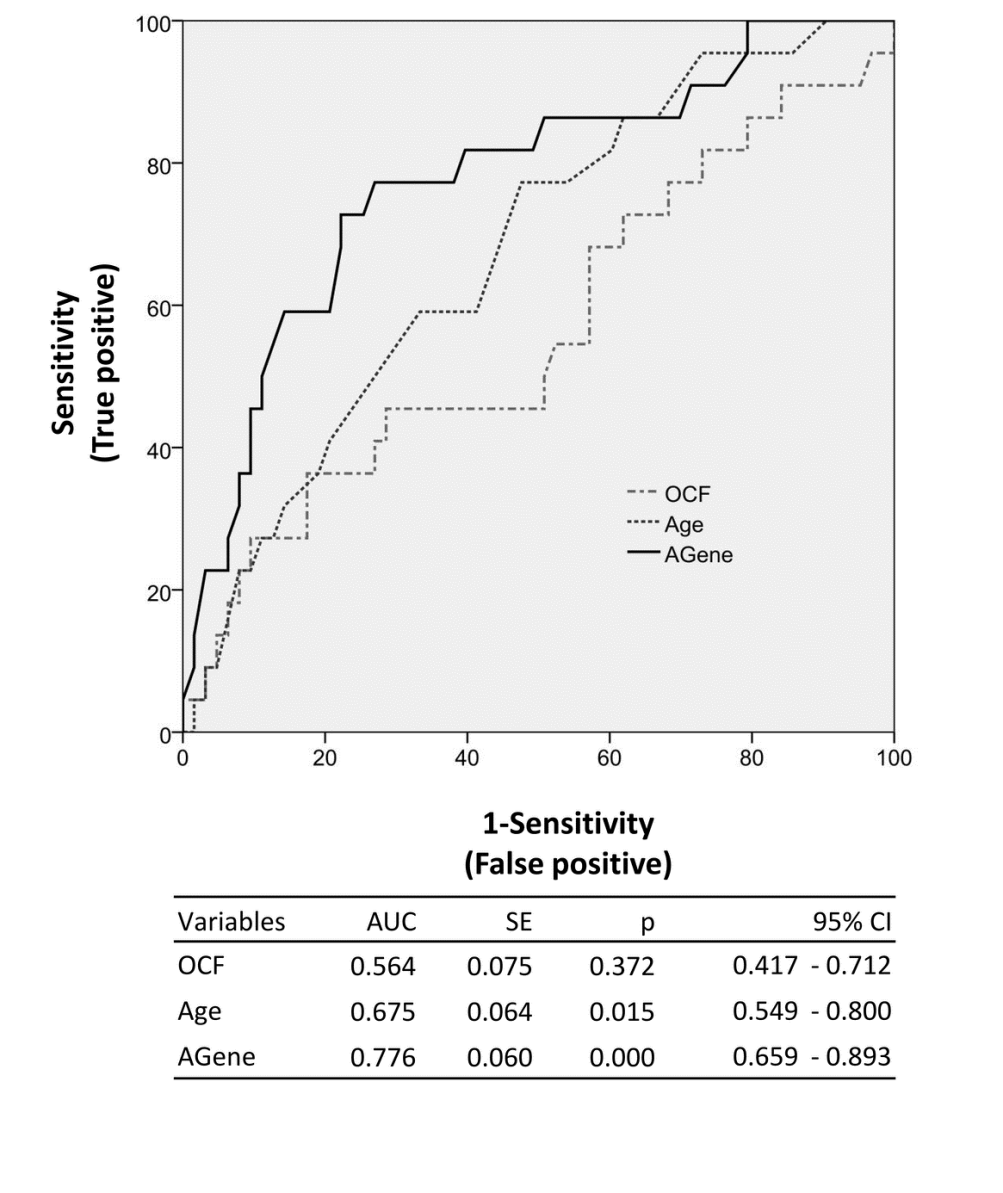
Receiver operating characteristic (ROC) analysis of OCF, Age and AGene in predicting RRR. OCF (other clinical factors) includes sex, surface area, performance status and smoking status. Agene includes age, rs2306283 and rs1051266. SE, standard error.

No linkage was observed among these three variants (Figure S2 in File S3). Haplotypic frequencies of *SLCO1B1* *1B, *1A and *15 were shown in Table S6 in File S2.

**Figure 3:**
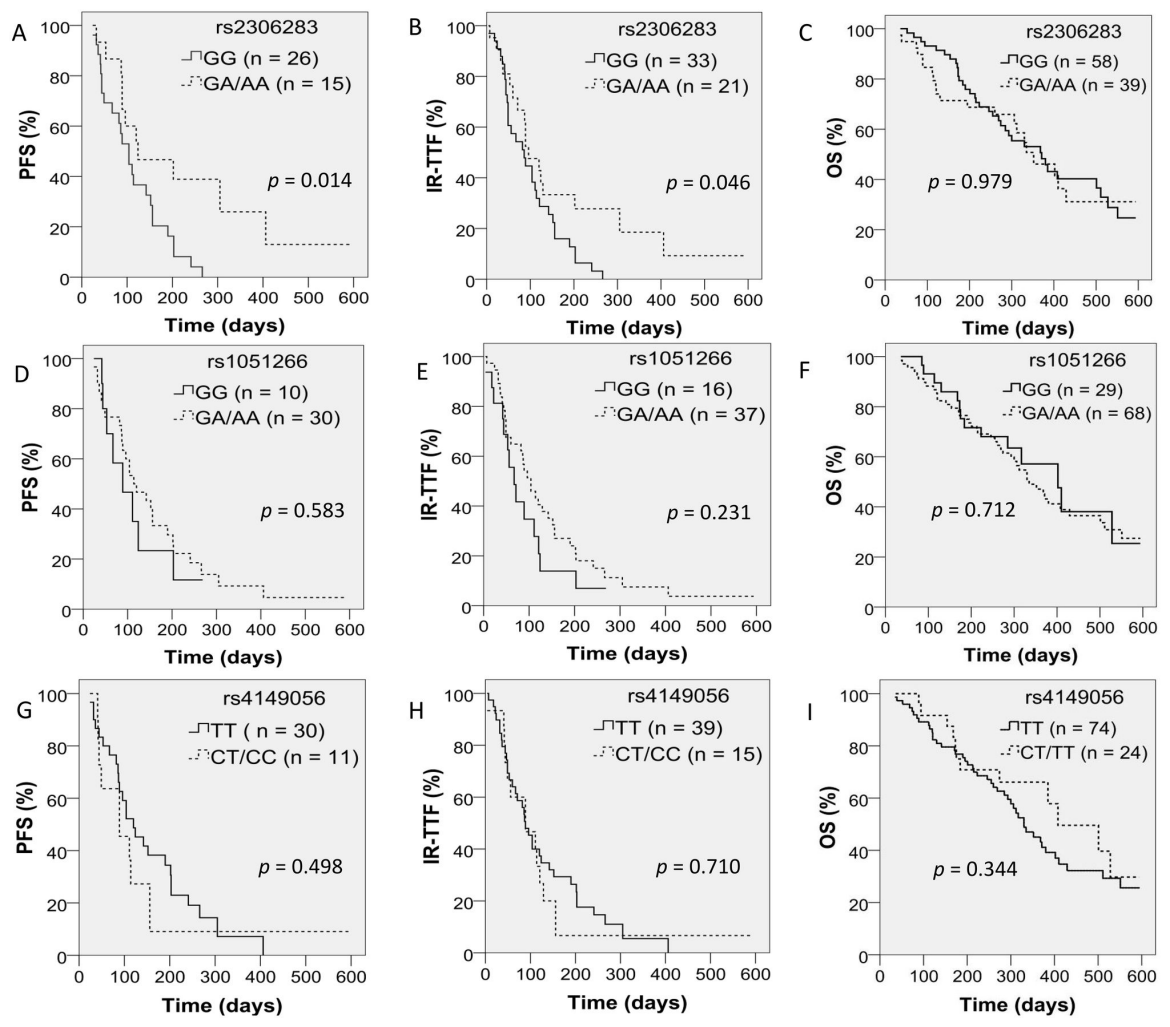
PFS (A, D, G), IR-TTF (B, E, H) and OS (C, F, I) in patients according to rs2306283, rs1051266 and rs4149056 genotypes (log-rank test).

**Table 4:** Multivariate logistic regression analysis (Enter) of RRR.

**Variate**	**RRR(N = 85)**		
	**OR**	**95% CI**	***P***
**rs2306283**			
GA/AA vs. GG	4.070	1.155-14.347	0.029
**rs1051266**			
GG vs. GA/AA	4.395	1.282-15.064	0.019
**rs4149056**			
CT/CC vs. TT	2.082	0.502-8.643	0.313
**sex**	0.861	0.141-5.243	0.871
**age**	1.072	1.013-1.135	0.017
**SA**	3.538	0.027-458.559	0.611
**PS**	1.014	0.945-1.087	0.701
**SS**	0.508	0.119-2.168	0.360

RRR, rapid response rate; N, No. of assessable patients; OR, odds ratio;

CI, confidence interval; SA, surface area; PS, performance status; SS, smoking status.

### Correlation between genotypes and response

The complete set of associations between each SNP and the RRR are shown in Table 3. Both rs2306283 (GA/AA) and rs1051266 (GG) were significantly associated with higher RRR by chi-squared test (Table 3) and univariate logistic regression analysis (Table S7 in File S2). As shown in table 3, RRR was 40% (14 of 35 patients) in rs2306283 (GA/AA) group compared to 15.7% (8 of 51 patients) in rs2306283 (GG) group, *p* = 0.011. In rs1051266 (GG) group, RRR was 45.8% (11 of 24 patients) compared to 19.4% (12 of 62 patients) in rs1051266 (GA/AA) group, *p* = 0.013. Patients with rs2306283 (GA/AA) and rs1051266 (GG) demonstrated a more significant difference in RRR (70% vs. 19.7%, OR = 9.489, *p* = 0.002). Differences were still significant even after correcting by Benjamini and Hochberg False Discovery Rate correction ( *p*_*c*_ < 0.05) and adjusting for potential clinical (sex, age, surface area, performance status, and smoking status) and genetic (rs4149056) covariates in multivariable logistic regression analysis (Table 4). In addition to genetic factors, age was the only clinical factor associated with RRR. To assess the contribution of the genetic factors to prediction of RRR, ROC analysis was performed using other clinical factors (OCF), age alone and age + rs2306283 + rs1051266 (AGene) respectively. AGene gave the highest AUC value (0.776, *p* < 0.001) and the best sensitivity (Figure 2), demonstrating that gene factors might improve predictive accuracy of RRR. 

### Correlation between genotypes and survival

At the median follow-up of 270 days (range 36–594 days), 69 patients had died, 3 patients did not come to their follow up appointment, and 125 patients had withdrawn from the study (53 experienced PD, 15 suffered intolerable grade ≥ 3 toxicities [1 patient withdrew because of rapid PD and intolerable toxicities], 41 patients without PD chose to give up the anti-cancer therapy, 15 non-PD patients accepted surgery or transcatheter arterial chemoembolization therapy, and 2 patients completed 12 cycles of chemotherapy without progression). In our study, the median OS was 343 days. To rule out that the differences in PFS and IR-TTF were due to non-chemotherapy factors, the PFS and IR-TTF evaluation excluded those patients with no medical reasons for withdrawal and non-PD patients who accepted an anti-cancer therapy other than chemotherapy. Thus, 41 and 54 patients were genotyped and analyzable for PFS and IR-TTF, respectively. Of whom, 1 patients failed to be genotyped for rs1051266. In Kaplan-Meier analysis, patients with rs2306283 (GA/AA) had significantly longer PFS (n = 41, median 124 vs. 104 days, log rank *p* = 0.014) and IR-TTF (n = 54, median 96 vs. 86 days, log rank *p* = 0.046) when compared with those with genotype GG. Similar results of univariate cox regression analysis of PFS, IR-TTF and OS were shown in Table S8 in File S2. In multivariate cox regression analysis, rs2306283 (GA/AA) again proved to be an independent prognostic factor of PFS (n = 40, HR = 0.402, 95% CI = 0.171–0.945, *p* = 0.037) but lose its significance in exhibiting a longer IR-TTF (n = 53, *p* = 0.076; Table 5). No association was observed between rs1051266 or rs4149056 and PFS or IR-TTF. None of the SNPs was a significant predictor of OS (n = 96, *p* > 0.05; Table 5). All survival curves for rs2306283, rs1051266 and rs4149056 are shown in Figure 3.

**Table 5:** Multivariate Cox regression analysis (Enter) of PFS, IR-TTF and OS.

**Variate**	**PFS(N = 40)**		**IR-TTF(N = 53)**		**OS(N = 96 )**	
	**HR(95%CI)**	***P***	**HR(95%CI)**	***p***	**HR(95%CI)**	***p***
**rs2306283**						
GA/AA vs. GG	0.402(0.171–0.945)	0.037	0.543(0.277-1.066)	0.076	0.966(0.536-1.741)	0.909
**rs1051266**						
GG vs. GA/AA	1.329(0.542-3.260)	0.534	0.714(0.358-1.423)	0.339	1.321(0.688-2.538)	0.403
**rs4149056**						
CT/CC vs. TT	1.197(0.527-2.718)	0.667	1.042(0.531-2.043)	0.905	0.688(0.341-1.390)	0.297
**sex**	0.724(0.234-2.238)	0.574	0.941(0.350-2.534)	0.905	0.893(0.363-2.201)	0.806
**age**	0.997(0.964-1.030)	0.852	1.008(0.981-1.035)	0.588	0.969(0.946-0.992)	0.008
**SA**	0.827(0.018-39.075)	0.923	1.298(0.058-29.145)	0.870	0.266(0.023-3.113)	0.292
**PS**	1.007(0.961-1.055)	0.771	1.006(0.969-1.045)	0.757	0.971(0.936-1.009)	0.130
**SS**	0.934(0.339-2.575)	0.895	1.079(0.475-2.452)	0.856	2.201(1.053-4.602)	0.036

PFS, progression-free survival; IR-TTF, irinotecan-related time to treatment failure; OS, overall survival; N, No. of assessable patients; HR, hazard ratio; CI, confidence interval; SA, surface area; PS, performance status; SS, smoking status.

## Discussion

We found that a common *SLC19A1* SNP, rs1051266 (GG) that was present in 29.9 % of our cohort, was associated with a higher FOLFIRI/mCapeIRI rapid response rate. To the best of our knowledge, this is the first prospective study to evaluate the effect of *SLC19A1* variants on FOLFIRI/mCapeIRI pharmacodynamics in mCRC patients. The SLC19A1 transporter is mainly localized at the apical brush-border membrane of the jejunum and colon and is regarded as the predominant route of folate uptake in mammalian cells[19]. Leucovorin (an activated form of folate) can help 5-FU to inhibit thymidylate synthase (TS) by forming a ternary complex with TS-FdUMP. Therefore, leucovorin-5-FU combinations have demonstrated a better therapeutic index than 5-FU alone in advanced colorectal cancer[29-32]. Low folate levels can increase the capability of leucovorin to form the complex[20]. Backus et al (2000) reported that four independent colon cancer cell lines cultured in low folate medium showed a higher sensitivity to either 5-FU alone or the combination with leucovorin[20]. Previous data have demonstrated that individuals with genotype rs1051266 (GG) had lower plasma folate levels[18]. The higher RRR of rs1051266 (GG) patients in our study may partially be explained by lower plasma folate and increased TS inhibition, but this explanation needs to be confirmed in further studies.

SLCO1B1 mediates the hepatic influx of SN38 from the blood. The other important finding is that rs2306283 (GA/AA) of *SLCO1B1* is associated with higher RRR, longer PFS and IR-TTF in mCRC patients treated by FOLFIRI/mCapeIRI regimens, while rs4149056 (CT/CC) failed to predict RRR or survival. However, previous studies have demonstrated that rs2306283 (A > G) may have little impact on SLCO1B1 activity [33,34] and no statistical significance was reported between rs2306283 and AUC_SN38_ or tumor response in 81 lung cancer patients[35], while the C allele of rs4149056 leads to decreased membrane expression of SLCO1B1, decreased transport activity, reduced drug clearance[36-39] and a higher plasma AUC_SN38_ in lung cancer patients[35]. A recent genome-wide association study among 699 children with acute lymphoblastic leukemia revealed that rs2306283 was associated with increased methotrexate (MTX, also substrates of SLCO1B1) clearance after adjusting for rs4149056[37]. Interestingly, our findings supported that rs2306283 (GA/AA) was independently associated with higher RRR, longer PFS and IR-TTF after adjusting for rs4149056. These findings demonstrate that in vitro or small, retrospective, single-institution studies usually have too many complex covariates to give conclusive results. Different ethnic background, different diseases, different regimens and different irinotecan doses may lead to controversial results. Renewed associations between rs2306283 (GA/AA) and treatment outcomes after adjusting for rs4149056 in prospective studies are worth pursuing in additional studies.

We also found that 70% of the patients with both rs2306283 (GA/AA) and rs1051266 (GG) achieved rapid response, much higher than other patients who were genotyped. To date, there is no functional data on the effects of combined *SLCO1B1* and *SLC19A1* gene variants in predicting response to FOLFIRI/mCapeIRI. The differences in RRR (70% vs. 19.7%), if validated, would provide valuable information for clinical decision-making. The predictive function of rs2306283 (GA/AA) combined with rs1051266 (GG), may help doctors figure out one subgroup of patients benefiting from conversion/neoadjuvant chemotherapy before surgery. But because of the small sample size, our findings need validation in larger series. 

None of the 3 SNPs was found to be associated with OS in our study, although several SNPs predicted a better response. This may be due to different subsequent treatments which may induce remission in patients resistant to first-line chemotherapy. In our study, the response rate is lower and the survival time is shorter than in previous clinical trials[6,24]. This may be because we have missed those patients who would have responded later than 12 weeks of chemotherapy because patients with stable disease received a lower median numbers of chemotherapy cycles (median number, 4; range, 2-12). In addition, the dropout rate before PD was high (34.2%) due to socioeconomic reasons. What’s more, only 5 patients (4.2%) accepted resection of liver metastases. These reasons may explain why good response was not related to longer OS in our study.

In conclusion, the results of this multi-center prospective study suggest that rs2306283 (GA/AA) of *SLCO1B1* in combination with rs1051266 (GG) of *SLC19A1* are significantly associated with rapid tumor response to FOLFIRI/mCapeIRI in Chinese mCRC patients. This may help doctors to optimize first-line chemotherapy of mCRC patients. Renewed associations between rs2306283 (GA/AA) and treatment outcomes are worth to pursue in the future. 

## Supporting Information

File S1 **Details of PCR reactions.**
(DOCX)

File S2 **Table S1 to S8.**
(DOCX)

File S3 **Figure S1 to S2.**
(DOCX)

Checklist S1 **TREND Checklist.**
(PDF)

Protocol S1 **Trial Protocol.**
(DOC)
