# Supplementary material for: SLCO1B1 and SLC19A1 Gene Variants and Irinotecan-Induced Rapid Response and Survival: A Prospective Multicenter Pharmacogenetics Study of Metastatic Colorectal Cancer
Source: PLoS One. 2013 Oct 15;8(10):e77223. doi: 10.1371/journal.pone.0077223 (PMC3797132; doi:10.1371/journal.pone.0077223)
Supplement: File S1 — Details of PCR reactions. (DOCX) [file pone.0077223.s001.docx]

**Details of PCR reactions**

PCR reactions (5 μl) were performed in standard 384-well plates using 10 ng genomic DNA, 0.5 U of Taqpolymerase (HotStarTaq, Qiagen,Valencia, CA), 0.1 μl of 25 mM dNTP, and 0.5 pM of each PCR primer. PCR thermal cycling was carried out for 4 min at 94℃, followed by 45 cycles of 20 s at 94℃, 30 s at 56℃, and 60 s at 72℃ and then 3 min at 72℃, 4℃ ∞. Shrimp Alkaline Phosphatase (0.5 U) (Sequenom, Hamburg, Germany) was added to the completed reactions and incubated for 20 min at 37℃, followed by inactivation for 5 min at 85℃. Post-PCR reactions were performed in a final volume of 9 μl of extension reaction containing 0.804 μl each Primer Mix (0.625–1.25mM), 0.041 μl enzyme, and 0.2 μl iPLEX Terminator (9–10 mM). PCR cycling was carried out for 30 s at 94℃, followed by 5 cycles of 5 s at 94℃, 5 s at 52℃, and 5 s at 80℃. The reaction procedure consisted of 40 cycles of denaturation at 94℃ and was completed by a final 3 min extension step at 72℃. The iPLEX reaction products were purified by 6 mg Clean Resin (Sequenom). The products were spotted on a SpectroChip (Sequenom), and data were processed and analyzed by MassARRAY TYPER 4.0 software (Sequenom).
