# Supplementary material for: SLCO1B1 and SLC19A1 Gene Variants and Irinotecan-Induced Rapid Response and Survival: A Prospective Multicenter Pharmacogenetics Study of Metastatic Colorectal Cancer
Source: PLoS One. 2013 Oct 15;8(10):e77223. doi: 10.1371/journal.pone.0077223 (PMC3797132; doi:10.1371/journal.pone.0077223)
Supplement: File S2 — Table S1 to S8. (DOCX) [file pone.0077223.s002.docx]

Table S1- Cancer centers in our study

| Name list |
| --- |
| Tongji Hospital, Tongji Medical College, Science and Technology of Huazhong University |
| Union Hospital, Tongji Medical College, Science and Technology of Huazhong University |
| Zhongnan Hospital, Wuhan University |
| Wuhan 8th Hospital, Wuhan, People’s Republic of China |
| Wuhan Pu-Ai Hospital, Tongji Medical College, Huazhong University of Science and Technology |
| Hubei Cancer Hospital |

Table S2- Criteria for irinotecan dose reduction

| Adverse effect | Grade | Dose reduction | | |
| --- | --- | --- | --- | --- |
|  |  | First time | Second time | Third time |
| Neutropenia or leukopenia | Ⅳ | Irinotecan reduce to 75%-80% | Irinotecan reduce to 50% | withdraw |
| Febrile neutropenia | Ⅲ |  |  |  |
| Thrombocytopenia and bleeding | Ⅳ | withdraw |  |  |
| Thrombocytopenia without bleeding | Ⅲ-Ⅳ | Irinotecan reduce to 75%-80% | Irinotecan reduce to 50% | withdraw |
| Non-hematologic toxicity (diarrhea, intestinal obstruction etc.) | Ⅳ | withdraw |  |  |
|  | Ⅲ | Irinotecan reduce to 75%-80% | Irinotecan reduce to 50% | withdraw |
| AST, ALT and serum creatinine meet the exclusion criteria and cannot be corrected in a week |  | withdraw |  |  |
| Bilirubin >1.5 - ≤ 3 times of upper limit of normal (ULN) |  | Irinotecan reduce to 50%-60% | withdraw |  |
| Bilirubin > 3 times of ULN |  | withdraw |  |  |

Table S3- Primer sequences

| SNP_ID | 2nd-PCRP | 1st-PCRP |
| --- | --- | --- |
| rs1051266 | ACGTTGGATGCGTAGAAGCAAAGGTAGCAC | ACGTTGGATGAGAAGCAGGTGCCCGTGGAA |
| rs 2306283 | ACGTTGGATGATTAAACAAGTGGATAAGG | ACGTTGGATGGATGTTCTTACAGTTACAGG |
| rs 4149056 | ACGTTGGATGGAATCTGGGTCATACATGTG | ACGTTGGATGTATGGGAGTCTCCCCTATTC |
| SNP_ID | UEP_DIR | UEP_SEQ |
| rs 1051266 | R | AGGTAGCACACGAGG |
| rs 2306283 | F | tttGATGTTGAATTTTCTGATGAAT |
| rs 4149056 | F | CTGGGTCATACATGTGGATATATG |
| SNP_ID | EXT1_SEQ | EXT2_SEQ |
| rs 1051266 | AGGTAGCACACGAGGC | AGGTAGCACACGAGGT |
| rs 2306283 | tttGATGTTGAATTTTCTGATGAATC | tttGATGTTGAATTTTCTGATGAATT |
| rs 4149056 | CTGGGTCATACATGTGGATATATGC | CTGGGTCATACATGTGGATATATGT |

2nd-PCRP, Secondary amplification primer (includes secondary tag); 1st-PCRP, Primary amplification primer (includes primer tag); UEP_DIR, Direction of MassEXTEND (F = Forward, R = Reverse); UEP_SEQ, extend primer sequence; EXT1_SEQ, sequence of analyte 1; EXT2_SEQ, sequence of analyte 2.

Table S4- Rapid response outcomes

| Effect | Response evaluable patients N (%) | | |
| --- | --- | --- | --- |
|  | GP | Non-GP | Total |
| rCR | 1 (1.1) | 0 | 1 (0.8) |
| rPR | 22 (25.3) | 10 (30.3) | 32 (26.7) |
| RRR | 23 (26.4) | 10 (30.3) | 33(27.5) |
| SD | 44 (50.6) | 15 (45.5) | 59 (49.2) |
| PD | 20 (23.0) | 8 (24.2) | 28 (23.3) |
| Total | 87 (88.8) | 33 (11.2) | 120 (100) |

rCR, rapid complete response; rPR, rapid partial response;

RRR, rapid response rate, RRR = rCR + rPR; SD, stable disease;

PD, progressive disease; GP, genotyped patients.

Table S5- Adverse events according to CTCAE 4.0

| Adverse events | Grade By CTCAE 4.0 N (%) | | | | | |
| --- | --- | --- | --- | --- | --- | --- |
|  | 0 | 1 | 2 | 3 | 4 | total |
| **Diarrhea** | 83(60.6) | 14(10.2) | 19(13.9) | 16(11.7) | 5(3.6) | 137(100%) |
| **Leukopenia** | 34(28.6) | 34(28.6) | 35(29.4) | 11(9.2) | 5(4.2) | 119(100%) |
| **Neutropenia** | 34(28.6) | 24(20.2) | 27(22.7) | 27(22.7) | 7(5.9) | 119(100%) |
| **Thrombocytopenia** | 105(88.2) | 6(5.0) | 5(4.2) | 3(2.5) | 0(0) | 119(100%) |
| **Vomiting** | 88(64.2) | 22(16.1) | 19(13.9) | 8(5.8) | 0(0) | 137(100%) |

Table S6 - Haplotypic frequencies of SLCO1B1

| **Haplotype** | **rs2306283** | **rs4149056** | **Frequency** |
| --- | --- | --- | --- |
| SLCO1B1*1B | G | T | 0.651 |
| SLCO1B1*1A | A | T | 0.217 |
| SLCO1B1*15 | G | C | 0.133 |

Table S7 - Univariate analysis (logistic regression) of RRR

| **Variate** | **N** | **OR** | **95% CI** | ***P*** |
| --- | --- | --- | --- | --- |
| **rs2306283** | **86** |  |  |  |
| GA/AA  GG | 35  51 | 3.583  1.000 | 1.301-9.871 | 0.014 |
| **rs1051266** | **86** |  |  |  |
| GG  GA/AA | 24  62 | 4.032  1.000 | 1.271-9.804 | 0.016 |
| **rs4149056** | **87** |  |  |  |
| TT  CT/CC | 67  20 | 1.000  0.907 | 0.288-2.858 | 0.868 |
| **Sex** | **120** |  |  |  |
| Male  Female | 77  43 | 1.000  0.711 | 0.301-1.680 | 0.438 |
| **SS** | **120** |  |  |  |
| Yes  No | 47  73 | 1.013  1.000 | 0.446-2.301 | 0.975 |
| **Age*** | **120** | 1.047 | 1.007-1.088 | 0.021 |
| **SA*** | **120** | 4.367 | 0.320-59.568 | 0.269 |
| **PS*** | **120** | 1.044 | 0.995-1.095 | 0.080 |

RRR, rapid response rate; N, No. of assessable patients;

OR, odds ratio; CI, confidence interval; SA, surface area;

PS, performance status; SS, smoking status. *: continuous variables

Table S8 - Univariate analysis (Cox regression) of PFS, IR-TTF and OS

| **Variate** | **N** | **PFS** | | **N** | **IR-TTF** | | **N** | **OS** | |
| --- | --- | --- | --- | --- | --- | --- | --- | --- | --- |
|  |  | **HR(95%CI)** | ***p*** |  | **HR(95%CI)** | ***p*** |  | **HR(95%CI)** | ***p*** |
| **rs2306283** | **41** |  |  | **54** |  |  | **97** |  |  |
| GA/AA vs. GG | 15  26 | 0.394  (0.181-0.856) | 0.019 | 21  33 | 0.539  (0.289-1.004) | 0.051 | 39  58 | 1.007  (0.586-1.732) | 0.979 |
| **rs1051266** | **40** |  |  | **53** |  |  | **97** |  |  |
| GA/AA vs. GG | 30  10 | 0.801  (0.359-1.786) | 0.588 | 37  16 | 0.682  (0.362-1.287) | 0.237 | 68  29 | 1.121  (0.611-2.056) | 0.713 |
| **rs4149056** | **41** |  |  | **54** |  |  | **98** |  |  |
| CT/CC  vs. TT | 11  30 | 1.288  (0.613-2.703) | 0.504 | 15  39 | 1.125  (0.600-2.108) | 0.713 | 24  74 | 0.736  (0.389-1.392) | 0.346 |
| **Sex** | **66** |  |  | **81** |  |  | **137** |  |  |
| Female vs. Male | 26  40 | 0.664  (0.373-1.184) | 0.165 | 31  50 | 0.726  (0.436-1.209) | 0.218 | 50  87 | 0.763  (0.469-1.243) | 0.278 |
| **SS** | **66** |  |  | **81** |  |  | **137** |  |  |
| Yes vs.  no | 20  46 | 1.464  (0.814-2.631) | 0.203 | 28  53 | 1.523  (0.922-2.516) | 0.100 | 53  84 | 1.687  (1.054-2.701) | 0.029 |
| **Age*** | **66** | 1.006  (0.982-1.030) | 0.647 | **81** | 1.011  (0.990-1.032) | 0.310 | **137** | 0.983  (0.964-1.002) | 0.084 |
| **SA*** | **66** | 1.863  (0.282-12.316) | 0.518 | **81** | 1.542  (0.286-8.308) | 0.614 | **137** | 0.466  (0.100-2.180) | 0.332 |
| **PS*** | **66** | 0.998  (0.969-1.029) | 0.912 | **81** | 0.998  (0.971-1.025) | 0.865 | **137** | 0.974  (0.947-1.002) | 0.069 |

N, No. of assessable patients; PFS, progression-free survival; IR-TTF, irinotecan-related time to treatment failure; OS, overall survival; HR, hazard ratio; CI, confidence interval; SA, surface area; PS, performance status; SS, smoking status.

*: continuous variables
