# Supplementary material for: SLCO1B1 and SLC19A1 Gene Variants and Irinotecan-Induced Rapid Response and Survival: A Prospective Multicenter Pharmacogenetics Study of Metastatic Colorectal Cancer
Source: PLoS One. 2013 Oct 15;8(10):e77223. doi: 10.1371/journal.pone.0077223 (PMC3797132; doi:10.1371/journal.pone.0077223)

Figure S1. PFS (A), IR-TTF (B) and OS (C) in patients with different chemotherapy regimens (log-rank test). No difference was found.


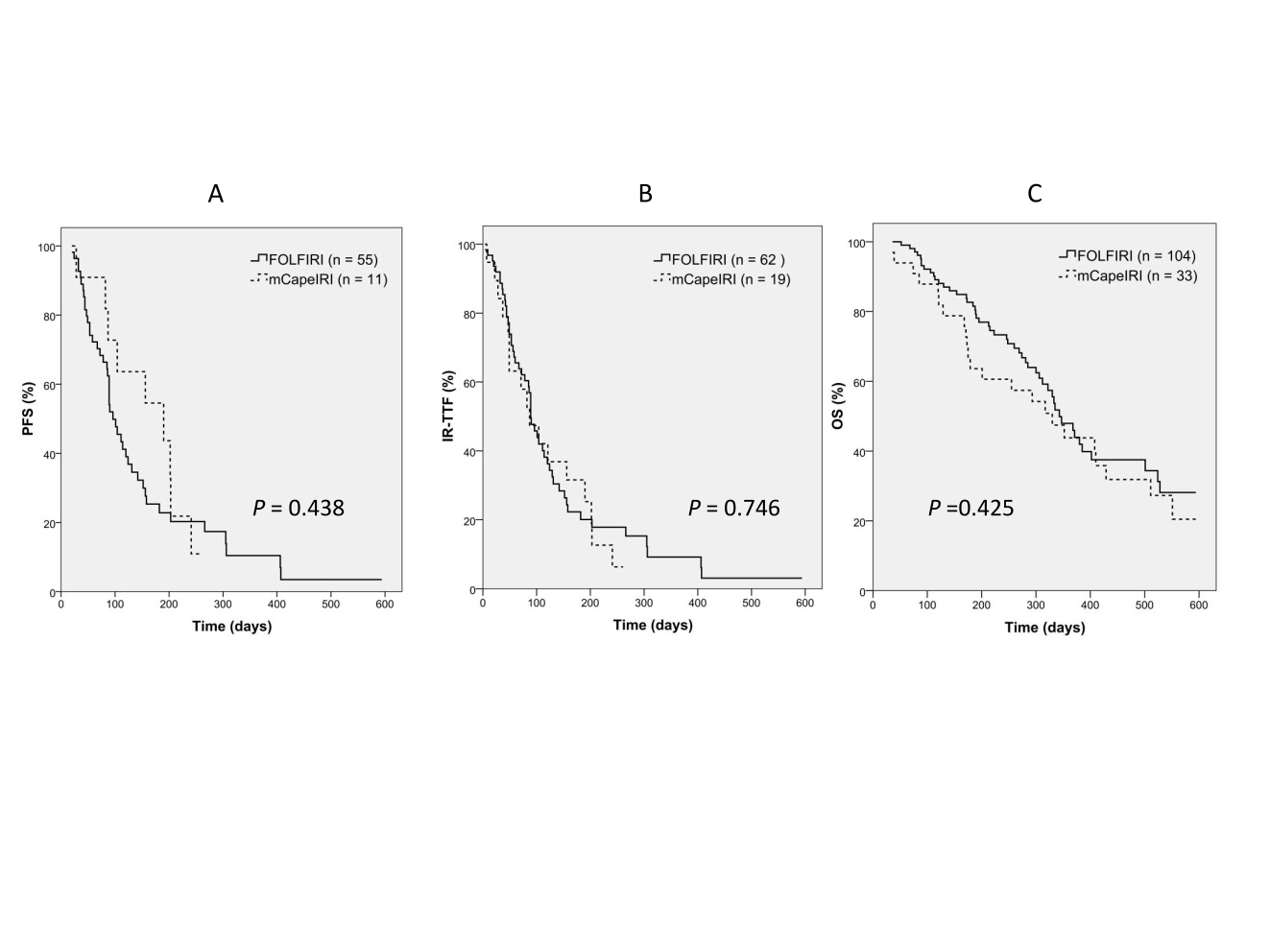


Figure S2. Linkage disequilibrium relationships among *SLCO1B1* (rs2306283, rs4149056) and *SLC19A1* (rs1051266) variants. The Lewontin’s coefficient D’ and the correlation coefficient r^2^ were reported.


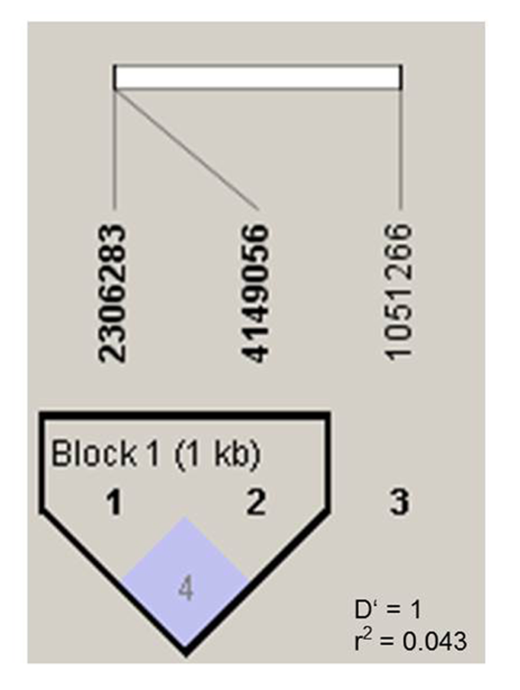

Supplement: File S3 — Figure S1 to S2. (DOCX) [file pone.0077223.s003.docx]
