# Supplementary material for: SLCO1B1 and SLC19A1 Gene Variants and Irinotecan-Induced Rapid Response and Survival: A Prospective Multicenter Pharmacogenetics Study of Metastatic Colorectal Cancer
Source: PLoS One. 2013 Oct 15;8(10):e77223. doi: 10.1371/journal.pone.0077223 (PMC3797132; doi:10.1371/journal.pone.0077223)
Supplement: Checklist S1 — TREND Checklist. (PDF) [file pone.0077223.s004.pdf]

TREND Statement Checklist

Paper Section/ Topic	Item No	Descriptor	Reported?	
				Pg #/Section
**Title and Abstract**				
Title and	1	Information on how unit were allocated to interventions		NA
Title and	1	Structured abstract recommended		Abstract
Title and	1	Information on target population or study sample		Material and methods
**Introduction**				
Background	2	Scientific background and explanation of rationale		Introduction
Background	2	Theories used in designing behavioral interventions		NA
**Methods**				
Participants	3	Eligibility criteria for participants, including criteria at different levels in recruitment/sampling plan (e.g., cities, clinics, subjects)		Patient eligibility and study design
Participants	3	Method of recruitment (e.g., referral, self-selection), including the sampling method if a systematic sampling plan was implemented		NA
Participants	3	Recruitment setting		Patient eligibility and study design
Participants	3	Settings and locations where the data were collected		Patient eligibility and study design
Interventions	4	Details of the interventions intended for each study condition and how and when they were actually administered, specifically including:		Treatment
Interventions	4	○ Content: what was given?		Treatment
Interventions	4	○ Delivery method: how was the content given?		Treatment
Interventions	4	○ Unit of delivery: how were the subjects grouped during delivery?		Treatment
Interventions	4	○ Deliverer: who delivered the intervention?		NA
Interventions	4	○ Setting: where was the intervention delivered?		Treatment
Interventions	4	○ Exposure quantity and duration: how many sessions or episodes or events were intended to be delivered? How long were they intended to last?		Treatment
Interventions	4	○ Time span: how long was it intended to take to deliver the intervention to each unit?		Treatment
Interventions	4	○ Activities to increase compliance or adherence (e.g., incentives)		Treatment
Objectives	5	Specific objectives and hypotheses		study design
Outcomes	6	Clearly defined primary and secondary outcome measures		study design
Outcomes	6	Methods used to collect data and any methods used to enhance the quality of measurements		study design and Efficacy and toxicity
Outcomes	6	Information on validated instruments such as psychometric and biometric properties		Genotyping
Sample Size	7	How sample size was determined and, when applicable, explanation of any interim analyses and stopping rules		study design
Assignment Method	8	Unit of assignment (the unit being assigned to study condition, e.g., individual, group, community)		Patient eligibility and study design
Assignment Method	8	Method used to assign units to study conditions, including details of any restriction (e.g., blocking, stratification, minimization)		NA
Assignment Method	8	Inclusion of aspects employed to help minimize potential bias induced due to non-randomization (e.g., matching)		NA

TREND Statement Checklist

Blinding (masking)	9	Whether or not participants, those administering the interventions, and those assessing the outcomes were blinded to study condition assignment; if so, statement regarding how the blinding was accomplished and how it was assessed.		NA
Unit of Analysis	10	Description of the smallest unit that is being analyzed to assess intervention effects (e.g., individual, group, or community)		NA
Unit of Analysis	10	If the unit of analysis differs from the unit of assignment, the analytical method used to account for this (e.g., adjusting the standard error estimates by the design effect or using multilevel analysis)		NA
Statistical Methods	11	Statistical methods used to compare study groups for primary methods outcome(s), including complex methods of correlated data		Statistical analysis
Statistical Methods	11	Statistical methods used for additional analyses, such as a subgroup analyses and adjusted analysis		Statistical analysis
Statistical	11	Methods for imputing missing data, if used		NA
Statistical	11	Statistical software or programs used		Statistical analysis
Results				
Participant flow	12	Flow of participants through each stage of the study: enrollment, assignment, allocation, and intervention exposure, follow-up, analysis (a diagram is strongly recommended)		Figure 1
Participant flow	12	o Enrollment: the numbers of participants screened for eligibility, found to be eligible or not eligible, declined to be enrolled, and enrolled in the study		Figure 1
Participant flow	12	o Assignment: the numbers of participants assigned to a study condition		Figure 1
Participant flow	12	o Allocation and intervention exposure: the number of participants assigned to each study condition and the number of participants who received each intervention		Figure 1
Participant flow	12	o Follow-up: the number of participants who completed the follow-up or did not complete the follow-up (i.e., lost to follow-up), by study condition		Figure 1
Participant flow	12	o Analysis: the number of participants included in or excluded from the main analysis, by study condition		Figure 1
Participant flow	12	Description of protocol deviations from study as planned, along with reasons		NA
Recruitment	13	Dates defining the periods of recruitment and follow-up		Patient characteristics and treatment outcome
Baseline Data	14	Baseline demographic and clinical characteristics of participants in each study condition		Patient characteristics
Baseline Data	14	Baseline characteristics for each study condition relevant to specific disease prevention research		NA
Baseline Data	14	Baseline comparisons of those lost to follow-up and those retained, overall		NA
Baseline Data	14	Comparison between study population at baseline and target population of interest		NA
Baseline equivalence	15	Data on study group equivalence at baseline and statistical methods used to control for baseline differences		Patient characteristics and treatment outcome

TREND Statement Checklist

Numbers analyzed	16	Number of participants (denominator) included in each analysis for each study condition, particularly when the denominators change for different outcomes; statement of the results in absolute numbers when feasible		NA
Numbers analyzed	16	Indication of whether the analysis strategy was “intention to treat” or, if not, description of how non-compliers were treated in the analyses		NA
Outcomes and estimation	17	For each primary and secondary outcome, a summary of results for each estimation study condition, and the estimated effect size and a confidence		Results
Outcomes and [illegible]	17	Inclusion of null and negative findings		Results
Outcomes and estimation	17	Inclusion of results from testing pre-specified causal pathways through which the intervention was intended to operate, if any		NA
Ancillary analyses	18	Summary of other analyses performed, including subgroup or restricted analyses, indicating which are pre-specified or exploratory		NA
Adverse events	19	Summary of all important adverse events or unintended effects in each study condition (including summary measures, effect size estimates, and confidence intervals)		Patient characteristics and treatment outcome
DISCUSSION				
Interpretation	20	Interpretation of the results, taking into account study hypotheses, sources of potential bias, imprecision of measures, multiplicative analyses,		Discussion
Interpretation	20	Discussion of results taking into account the mechanism by which the intervention was intended to work (causal pathways) or alternative mechanisms or explanations		Discussion
Interpretation	20	Discussion of the success of and barriers to implementing the intervention,		NA
Interpretation	20	Discussion of research, programmatic, or policy implications		NA
Generalizabilit y	21	Generalizability (external validity) of the trial findings, taking into account the study population, the characteristics of the intervention, length of follow-up, incentives, compliance rates, specific sites/settings involved in the study, and other contextual issues		Discussion
Overall Evidence	22	General interpretation of the results in the context of current evidence and current theory		Discussion

From: Des Jarlais, D. C., Lyles, C., Crepaz, N., & the Trend Group (2004). Improving the reporting quality of nonrandomized evaluations of behavioral and public health interventions: The TREND statement. *American Journal of Public Health*, 94, 361-366. For more information, visit: http://www.cdc.gov/trendstatement/
