# Supplementary material for: SLCO1B1 and SLC19A1 Gene Variants and Irinotecan-Induced Rapid Response and Survival: A Prospective Multicenter Pharmacogenetics Study of Metastatic Colorectal Cancer
Source: PLoS One. 2013 Oct 15;8(10):e77223. doi: 10.1371/journal.pone.0077223 (PMC3797132; doi:10.1371/journal.pone.0077223)
Supplement: Protocol S1 — Trial Protocol. (DOC) [file pone.0077223.s005.doc]

Pharmacogenomics Study of CPT-11 as the First-line Chemotherapy for Metastatic Colorectal Cancer

1. **Background**

Colorectal cancer (CRC) is the third leading cause of cancer-related deaths in China and leads to nearly 180,000 deaths per year. Irinotecan (CPT-11) combined with 5-FU/LV (FOLFIRI) is recommended as one of the first-line chemotherapy regimens for Metastatic Colorectal Cancer (mCRC).The variation seen in patient response and tolerability remains a clinical challenge. Some clinical studies showed a significant relationship between the gene polymorphisms in key enzymes (eg.UGT1A1, ABCB1, SLCO1B1 etc) involved in the metabolism of CPT-11 and its toxicity. But there are three limitations. Firstly, most of the studies were either retrospective analysis or with small sample size and some of the results were conflict. Secondly, Chinese were rarely included. Thirdly, most of them only used candidate gene approach. By using prospective method, in our study, a larger sample will be enrolled and our aim is to find reliable genetic markers for predicting clinical outcome and toxicity in mCRC patients treated with CPT-11 chemotherapy.

When diagnosed with colorectal cancer, 35% of those patients are stage Ⅳ. Approximately 50%-60% of patients diagnosed with colorectal cancer will develop metastases. Irinotecan is a camptothecin analogue with strong antitumor activity through inhibition of topoisomerase I. Objective response rate (ORR) is about 35%-50%.Compared with treatment with 5-FU/LV, treatment with irinotecan, fluorouracil, and leucovorin resulted in significantly longer progression free survival, higher rate of confirmed response, and longer overall survival. Toxicities associated with irinotecan include both early and late forms of diarrhea (Grade3 to 4 are 22%-30%), and neutropenia (Grade3 or 4 are about 50%). The V308 study had demonstrated that FOLFIRI followed by FOLFOX6 led to longer median Progression-free survival (PFS) and better tolerability in second-line therapy than the reverse sequence.[^6^](#_ENREF_6) Due to being afraid of the toxicity (e.g. fatal diarrhea) of CPT-11，most doctors in China would like to choose FOLFOX instead of FOLFIRI.

Patients respond differently to identical medications. How to match the right drug to the right person? Nowadays, personalized therapy based on the genotype represents the future direction in cancer treatment.

**
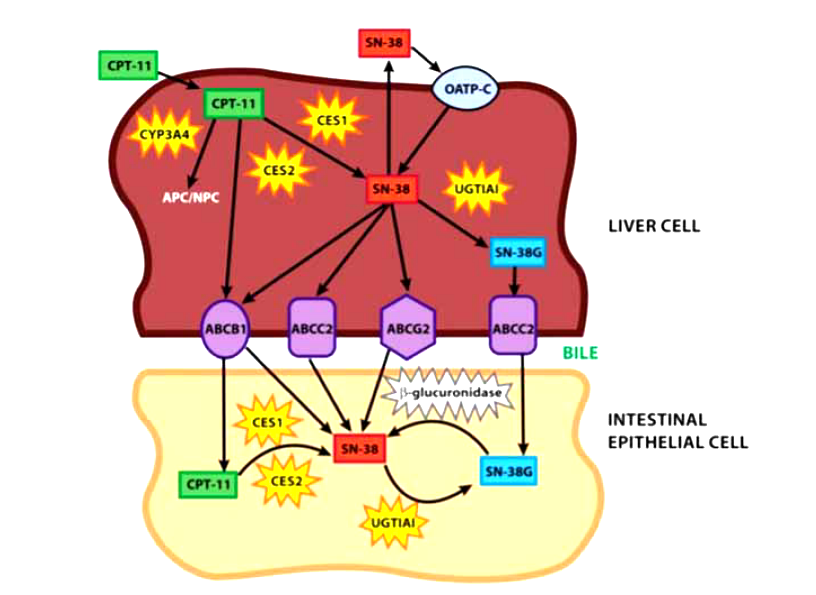
**

Fig. 1: **The metabolism of CPT-11** .[^7^](#_ENREF_7)

CPT-11 is converted to its pharmacologically active metabolite, SN-38 by carboxylesterase1 (CES1) or carboxylesterase2 (CES2) which are both expressed in gastrointestinal tissue, more specifically, within the enterocytes, as well as in the liver. It has been shown that CES2 has a 12.5- to 26-fold higher affinity for CPT-11 than that of CES1.

Three elimination pathways exist for SN-38. First, it can be further conjugated and detoxified by the UDP-glucurono-syltransferase (UGT) enzyme to the ß-glucuronic acid conjugate (SN-38G). Secondly, SN-38 can be eliminated by membrane-localized, energy-dependent outward drug pumps, which facilitate cellular efflux mechanisms. Some of these pumps include proteins belonging to the super family of ABC transporters. One other important transporter involved is the hepatic organic anion transporter (SLCO1B1). These protein transporters are responsible for the biliary and intestinal excretion of CPT-11, and to a lesser degree, of SN-38, and SN-38G. Thirdly, CPT-11 may be eliminated before hydrolysis to its active form by the CYP3A4 enzyme, a member of the cytochrome P450 super family.

CPT-11 functions as an inhibitor of topoisomerase I (Topo1), an enzyme involved in the unraveling of the DNA helix during replication.

Pharmacogenetics, a subset of pharmacogenomics, is a study of genetically determined alterations in metabolic pathways of drug. We have already known some pharmacogenetics information about CPT-11 and most of them originated from small sample study by candidate gene approach. Some of candidate genes are not included in previous studies.

For example, studies has shown that the combination of at least one SLCO1B1 521 T allele, one ABCB1 1236 C allele and one UGT1A1*28 variant 7 repeat demonstrated a statistically significant association with grade 3/4 toxicities in mCRC patients.[^7^](#_ENREF_7) UGT1A1genotype are strongly associated with severe neutropenia and diarrhea. But the genotype was not related to tumor response or to overall survival (OS). [^10^](#_ENREF_10) However, Carlini LE’s research showed that UGT1A7 and/or UGT1A9 genotypes may be predictors of response and toxicity in CRC patients treated with capecitabine plus irinotecan.[^11^](#_ENREF_11) ABCB1 and ABCG2C polymorphism demonstrates different CPT-11 drug exposure.[^12^](#_ENREF_12) Cytochrome P450 (CYP) polymorphism (e.g.CYP2D6, CYP2C19), was a major source of the heterogeneity in drug exposure. Still there are even more we don’t know yet.

What’s more, in different populations, genotype variants are more or less frequent. Remarkable interethnic differences in the distributions of UGT1A1 genetic polymorphisms have been known. The allele frequency of UGT1A1*28 among Caucasians is higher (0.3 to 0.4) than that among Asians (0.1 to 0.2). On the contrary, other variant alleles in the coding regions were found only in Asians.[^13^](#_ENREF_13)

Does Topo1 immunohistochemistry have any effect on the clinical response or prognosis for colorectal cancer patients treated with 5-FU/CPT-11 regimen? It is debated now. Some studies showed that it does not differ in TS or Topo-1 expression level in tumor.[^14^](#_ENREF_14) But some indicated that patients with moderate/high level of Topo-1 benefited from CPT-11.[^15^](#_ENREF_15) How about Topo1 gene polymorphism? Hoskins demenstrated that TOP1 and TDP1 htSNPs were related to grade 3/4 neutropenia (P = 0.04) and response (P = 0.04), respectively. [^16^](#_ENREF_16)But that’s not enough.

For the three limitations mentioned in the preface part, we think it is essential to carry out a prospective study in the relationship between the irinogenetics and CPT-11 toxicity, as well as the effectiveness or clinical outcome in China.

1. **Candidate genes**

- Topo1
- Carboxylesterase (CES)
- CYP3A4 cytochrome P450, family 3, subfamily A, polypeptide 4
- UGT1A1
- [ATP-binding cassette (ABC) transporters](http://en.wikipedia.org/wiki/ATP-binding_cassette_transporter)

1. **Objective and endpoints**

**Objective:** To determine the correlation between irinogenetics and irinotecan -related severe toxicities or tumor response in Chinese mCRC patients.

**Primary endpoints:** The correlation between SNP and ORR. The correlation between SNP and adverse events of Grade ≥3 (blood toxicities, diarrhea and any other serious adverse event).

**Secondary endpoints:** The correlation between SNP and PFS/OS.

1. **Research design**

Patients who meet the inclusion criteria 1-12

Record the clinical message

Collect blood samples after the consent of patients

Evaluation of target lesions

AE/toxicity

eg: diarrhe

neutropenia ect.

OS

PFS

Blood samples of each patient

Genoe-wide association study or relavant candidate gene study

The associations of each polymorphism with tumor response, toxicity, and clinical outcome (survival) will be analyzed.

1. **Method**

**Relavant candidate gene study**

Gene Oncology ([http://www.geneontology.org](http://www.geneontology.org/)) and the National Center for Biotechnology Information (NCBI) Gene database (<http://www.ncbi.nlm.nih.gov/gene>) and related literature to identify all functional single nucleotide polymorphisms (SNPs) of relavant genes with a minor allele frequency of more than 0.15 and a location in the promoter untranslated region or coding region of the gene.

**Genome-wide association study**

The GWAS will be conducted using an Affymetrix Genome-Wide Human SNP Array 6.0, followed by a systematic quality control step before the association analysis.

1. **Inclusion and Exclusion criteria**

**6.1 Inclusion criteria**

1. Histologically confirmed unresectable adenocarcinoma of colorectum
2. ≥ 18 years old
3. measurable disease, defined according to the Response Evaluation Criteria In Solid Tumors version 1.1 (RECIST1.1)
4. Unresectable metastatic disease or first recurrence/metastasis after adjuvant therapy and not suitable for operation
5. Without expected course of radiotherapy during the first-line chemotherapy
6. No previous CPT-11 exposure
7. ECOG performance status (PS) 0-2
8. Not pregnant or nursing
9. Voluntarily signed the informed consent
10. Total bilirubin ≤ 1.5 times upper limit of normal (ULN)
11. AST and ALT ≤ 2.5 times ULN (≤ 5 times ULN if liver metastases present)
12. Creatinine clearance > 50 ml/min or serum creatinine ≤ 1.5 times ULN

**6.2 Exclusion criteria**

1. Brain metastases
2. Severe bone marrow failure and cannot be corrected
3. Chronic diarrhea history
4. Bowel obstruction without control
5. Mental illness without control
6. Clinically significant (i.e., active) cardiovascular disease, including any of the following: Cerebrovascular accidents/Myocardial infarction/Unstable angina/ New York Heart Association class II-IV congestive heart failure/Serious cardiac arrhythmia requiring medication/Uncontrolled hypertension
7. Other co-existing malignancy or malignancy diagnosed within the past 5 years, except for basal cell or squamous cell carcinoma, or carcinoma in situ of the cervix
8. Pelvic radiotherapy in the past 1 year
9. Known allergy to any of the components of the study medications
10. Serious, no healing wound or ulcer

**6.3 Withdrew criteria**

1. Disease progression
2. Intolerable toxicities
3. Patients asked to withdrew from study due to any reason
4. Loss of follow-up
5. Death due to any cause
6. **7. Chemotherapy regimen and dose modifications**

**7.1 Chemotherapy regimen**

FOLFIRI

Irinotecan 180 mg/m^2^ IV over 30-90 minutes, day 1

Leucovorin 400 mg/ m^2^ IV infusion to match duration of irinotecan infusion, day 1

5-FU 400 mg/ m^2^ IV bolus on day 1, then 1200 mg/ m^2^ /day x 2 days (total 2400 mg/ m^2^ over 46-48 hours) continuous infusion

Repeat every 2 weeks (14 days are one cycle) until PD or intolerable toxicities.

The starting dose of irinotecan is 180mg/m2 and then it will be reduced lower than 180mg/m^2^ according to dose modification table.

**7.2 Dose modifications table 1.**

| Adverse effect | Grade | Dose reduction | | |
| --- | --- | --- | --- | --- |
|  |  | First time | Second time | Third time |
| Neutropenia or leukopenia | Ⅳ | Irinotecan reduce to 75%-80% | Irinotecan reduce to 50% | withdraw |
| Febrile neutropenia | Ⅲ |  |  |  |
| Thrombocytopenia and bleeding | Ⅳ | withdraw |  |  |
| Thrombocytopenia without bleeding | Ⅲ-Ⅳ | Irinotecan reduce to 75%-80% | Irinotecan reduce to 50% | withdraw |
| Non-hematologic toxicity (diarrhea, intestinal obstruction etc.) | Ⅳ | withdraw |  |  |
|  | Ⅲ | Irinotecan reduce to 75%-80% | Irinotecan reduce to 50% | withdraw |
| AST, ALT and serum creatinine meet the exclusion criteria and cannot be corrected in a week |  | withdraw |  |  |
| Bilirubin >1.5 - ≤ 3 times of upper limit of normal（ULN） |  | Irinotecan reduce to 50%-60% | withdraw |  |
| Bilirubin > 3 times of ULN |  | withdraw |  |  |

**Note：**

1. For patients who had prior pelvic radiotherapy within one year before chemotherapy, CPT-11 was reduced to 75% to 80%
2. Hematologic toxicity needs to recovered to ≤ Grade 1 before chemotherapy
3. Clinical standard operating procedure (SOP) of chemotherapy delay due to toxicities

Normal schedule d1→d8→d15→d22→d29→d36→d43→d50→d57

Delayed < 1week d1→d8→d15→d18→d29→d32→d36

Delayed > 1week d1→d8→d15→d22→d25→d29→d36→

副反应恢复

1. **Tests before chemotherapy**

Blood count

Blood biochemical (15 liver function, kidney function 4, two of lipids, electrolytes four fasting glucose, LDH)

Routine urine

Time for tests: chemotherapy ± 3 days

CT/MRI for baseline evaluation

1. **Tumor response evaluation**

According to RECIST1.1, evaluate every 6 weeks’ chemotherapy.

1. **Adverse effects evaluation**

National Cancer Institute Common Toxicity Criteria 4.0

1. **Staging**

AJCC 7^th^ edition (2010) TNM staging system for colon/rectal cancer

1. **Sample size calculation and Statistical method**

12.1 Sample size calculation

**Candidate gene study**

we choose type I error α = 0.05, 1-β = 0.8, two-sided test, provided the target SNP allele frequency in the population was about 20%, treatment efficacy was about 30%, OR ≥ 3.5, the calculated samples size was ≤ 86.

**GWAS study**

We set statistical limits for the type I error α = 0.01, 1-β = 0.8, two-sided test, provided the target SNP frequency in the population > 15%, and OR> 4, according to the known lowest FOLFIRI first-line treatment induced severe adverse reactions (about 20%) and treatment efficacy (about 30%), we calculate that the needed samples will be 112 by software Quanto (Version 1.2.4), 1:1 ratio.

12.2 Statistical method

Hardy-Weinberg equilibrium will be assessed. The χ2 test will be used to compare the observed genotype frequencies with those expected under Hardy-Weinberg equilibrium.

The associations of each polymorphism with tumor response, toxicity, and demographic variables were examined using contingency tables and the Fisher’s exact test. Multivariate Logistic Regression will be performed to ascertain which genotype independently predicted response or toxicity. Odds ratios (OR) and their 95% confidence interval (CI) were calculated as estimates of the correlations. Kaplan–Meier estimates the PFS and OS. To evaluate the relationship between genotypes and PFS, IR-TTF, and OS, multivariate Cox regression (forward LR) performed, adjusting for potential confounding covariates. Hazard ratios (HR) and their 95% CI were calculated as estimates of the correlations. A value of *p* < 0.05 was considered statistically significant in a two-tailed test.

Statistical analyses were performed using SPSS16.0 (SPSS Inc, Chicago, IL) and two-sided P-value of 0.05 is considered significant.

1. **Research institutions**

Sponsor

- Tongji Hospital, Tongji Medical College, Science and Technology of Huazhong University

Collaborators

- Union Hospital, Tongji Medical College, Science and Technology of Huazhong University
- Zhongnan Hospital, Wuhan University
- Wuhan 8th Hospital, Wuhan, People’s Republic of China
- Wuhan Pu-Ai Hospital, Tongji Medical College, Huazhong University of Science and Technology
- Hubei Cancer Hospital
